# Prolonged postoperative length of stay may be a valuable marker for susceptibility to relapse beyond established risk factors in patients with stage III colon cancer

**DOI:** 10.1186/s12957-022-02742-8

**Published:** 2022-09-02

**Authors:** Frans Emland, Helena Taflin, Göran Carlsson, David Ljungman, Elinor Bexe Lindskog

**Affiliations:** 1 Sahlgrenska Academy at University of Gothenburg, Box 400, 405 30, Gothenburg, Sweden; 2 Department of Surgery, Institute of Clinical Sciences, Sahlgrenska Academy, Sahlgrenska University Hospital/Transplant Centre, University of Gothenburg, Gothenburg, Sweden; 3 Department of Surgery, Institute of Clinical Sciences, Sahlgrenska Academy, University of Gothenburg, Gothenburg, Sweden; 4 Region Västra Götaland, Department of Surgery, Sahlgrenska University Hospital, 416 85 Gothenburg, Sweden

**Keywords:** Colonic neoplasm, Postoperative length of stay, Prognostic risk factor, Disease-free survival

## Abstract

**Background:**

Delay from surgery to adjuvant chemotherapy causes impaired survival among patients undergoing radical resection for stage III colon cancer, and the underlying mechanism for this is incompletely clarified. It is established that prolonged postoperative hospital length of stay (LOS) is associated with delayed initiation of the adjuvant treatment driving the assumption that prolonged LOS is prognostically unfavorable due to this fact and case mix factors. We hypothesize that prolonged LOS after surgery is a valuable marker for susceptibility to relapse that is not detected in established prognostic factors and, alone, associated with a shorter disease-free survival (DFS).

**Materials and methods:**

A total of 690 consecutive patients undergoing elective radical resection for stage III colon cancer in 2000–2015 were identified in a prospective detailed facility database. Univariate and multivariate analyses were performed using Cox proportional hazards model in the evaluation of LOS as an independent prognostic factor.

**Results:**

Short postoperative LOS, low comorbidity, and few complications were associated with longer DFS (*p* < 0.01). Fewer patients in the short and intermediate LOS groups had a relapse in their disease (28% and 33%, respectively), compared to the patients with longer LOS (40%, *p* < 0.05). LOS was a prognostic factor for DFS in the unadjusted univariate model (HR 1.04 per unit change) and remained statistically significant in the adjusted multivariate analysis, with a HR of 1.03 per hospital day (*p* < 0.01).

**Conclusions:**

Postoperative LOS independently correlates with the risk of recurrence and DFS, regardless of if adjuvant chemotherapy is given, along with the factors such as age, comorbidity, complications, and tumor features. We propose a further investigation into the causal mechanisms based on tumor and host biology linking LOS to DFS beyond established risk factors.

**Supplementary Information:**

The online version contains supplementary material available at 10.1186/s12957-022-02742-8.

## Background

Colorectal cancer (CRC) is the third most common form of cancer in the world, and around two-thirds of these consist of colon cancer [1]. Surgery remains the basis for curative treatment, while chemotherapy is recommended for all patients with stage III colon cancer without contraindications to reduce the risk of recurrence after radical surgery [2, 3]. The benefit of adjuvant treatment still varies within stage III, emphasizing the importance of individual risk stratification [3, 4].

Both enhanced recovery care and laparoscopy have become the crucial parts of the perioperative care of patients with colon cancer, consequently reducing the postoperative LOS [5–7]. The strongest predictive factors for a prolonged LOS are postoperative reoperations, infections at the surgical site, and open surgery in contrast to laparoscopic surgery [8].

Adjuvant chemotherapy should be initiated close in time to surgery and at the latest within 8 weeks for the best effect [3, 9, 10]. A contributing factor to delayed adjuvant chemotherapy is prolonged LOS, consequently associating prolonged LOS with a worse outcome [11].

Delayed adjuvant chemotherapy has in many studies been shown to shorten both DFS and overall survival (OS) [9, 10]. It is yet, to our knowledge, not known if prolonged LOS alone correlates with the oncological outcome. While just a few studies have been carried out on this issue, findings are hampered by the incoherent composition of the cohorts: studying colon and rectal cancer patients, mixed stages, and with no relation to adjuvant chemotherapy [12, 13].

The rationale behind this study lies within the fact that delayed adjuvant chemotherapy is prognostically unfavorable, while the underlying mechanism for this is incompletely clarified. Tumor immunological factors affecting minimal residual disease in patients treated for stage III colon cancer could be hidden in the LOS, encouraging the present study to evaluate if prolonged LOS is of prognostic value.

Tumor biology is of great importance for prognosis and survival but has little impact on decisioning on adjuvant chemotherapy. In the clinical setting age, comorbidity, postoperative convalescence, and by extension LOS are the main factors determining if, and when, a patient is eligible for adjuvant chemotherapy. A postponed postoperative follow-up, caused by prolonged LOS, risks ruling out or delaying the adjuvant chemotherapy. The aim of the present study is to investigate how postoperative hospital LOS independently impacts the prognosis of patients treated for stage III colon cancer, in relation to adjuvant chemotherapy.

## Material and methods

### Study design and population

All patients in this study have undergone surgery for stage III colon cancer at Sahlgrenska University Hospital (SU) between the years 2000 and 2015. We included only stage III to have a homogenous cohort where the group at large is recommended adjuvant chemotherapy. The inclusion criteria for participating in the study were elective surgery, microscopic radical resection, and at least 90 days of postoperative survival. The study period was set to include the 5-year follow-up; 6 patients were lost to follow-up and thereby excluded. Full analyses were performed in a cohort of 690 patients. Length of stay (LOS) was defined as the number of days spent in the hospital from the day of surgery until discharge.

The endpoint in the study was disease-free survival (DFS), defined as the time from radical surgery to an event. Local or distal recurrence, all-cause mortality, or new CRC were all classified as events. Patients were censored if lost to follow-up or living without disease at the end of the study period. DFS was favored above other outcome measures, due to the nature of this study and considering the variables being investigated. The overall survival (OS) includes more confounding factors and more to take into consideration, for example, whether a patient with recurrence receives first-, second-, or third-line chemotherapy, as well as if the patient goes through new potentially curable surgery.

Comorbidity, postoperative complications, LOS, any reinterventions, and readmissions were collected through medical records. Comorbidities were assessed by the age-adjusted Charlson Comorbidity Index (ACCI) [14] and postoperative complications according to the Clavien-Dindo (CD) classification [15]. Using ACCI, every decade above 40 years is given 1 point; hence, age is adjusted in the final analysis. ACCI were grouped into low (0–2 points), intermediate (3–5 points), and high (6–9 points). Clavien-Dindo was dichotomized into minor (grade 0–3a) and major (grade 3b–4) complications.

### Statistical analysis

All statistical analyses were carried out by the statistical software JMP®, version 15.0.0, for Macintosh (SAS Institute Inc., Cary, NC). The significance level was set to a two-sided *p*-value of < 0.050. As quantitative data, such as age, had a skewed distribution, they are presented with a median value together with the minimum and maximum value or the measure of variability, interquartile range (IQR), and statistical significance tested with the nonparametric Wilcoxon test. Qualitative variables at the nominal level, for example, tumor location, are presented as frequencies and percentages, and Pearson’s chi-squared test was used for significance testing.

Univariate analyses were carried out for specific parameters, using Cox proportional hazards model to assess which factors were influencing the DFS. A multivariate model was constructed, with adjustment for possible confounders in the assessment of LOS as an independent prognostic factor for DFS.

## Results

In the entire cohort of 690 patients, the median age was 71 years, and a narrow majority of patients were female (54%). Patients were distributed as evenly as possible into three groups according to their LOS, in order to analyze how different clinical characteristics were associated with a longer LOS: shorter (≤ 5 days), intermediate (6–8 days), and prolonged (> 8 days) postoperative hospital stay. A full description of the clinical characteristics of the three LOS cohorts is presented in Table 1.

**Table 1:** Clinical characteristics of stage III colon cancer patients by hospital length of stay cohorts

Variables	Length of stay (days)			***p***-value	Total (***n*** = 690)
	≤ 5 (***n*** = 202)	6–8 (***n*** = 248)	> 8 (***n*** = 240)		
**Length of stay (days)**					
Median (min-max)	5 (3–5)	7 (6–8)	12 (9–51)		7 (3–51)
Mean	4.5	6.9	14.5		8.8
**Age, median (min-max)**	69 (27–92)	69 (19–91)	73 (19–93)	< 0.05	71 (19–93)
**Gender,*n*(%)**					
Female	106 (52)	145 (58)	124 (52)	NS	375 (54)
Male	96 (48)	103 (42)	116 (48)		315 (46)
**Surgery,*n*(%)**^a^					
Open	142 (70)	221 (89)	222 (93)	< 0.01	585 (85)
Laparoscopic	60 (30)	27 (11)	18 (8)		105 (15)
**Tumor location,*n*(%)**^a^					
Right sided	113 (56)	134 (54)	121 (50)	NS	368 (53)
Left sided	88 (44)	113 (46)	112 (47)		313 (45)
Multiple locations	1 (1)	1 (0)	7 (3)		9 (1)
**Differentiation grade,*n*(%)**^a^					
Low grade (G1–G2)	142 (70)	172 (69)	169 (70)	NS	483 (70)
High grade (G3–G4)	47 (23)	63 (25)	58 (24)		168 (24)
Mucinous	13 (6)	13 (5)	13 (5)		39 (6)
**T-stage,*n*(%)**^a^					
1	5 (2)	2 (1)	3 (1)	NS	10 (1)
2	14 (7)	16 (6)	14 (6)		44 (6)
3	150 (74)	178 (72)	159 (66)		487 (71)
4	33 (16)	52 (21)	64 (27)		149 (22)
**N-stage,*n*(%)**					
N1	136 (67)	152 (61)	158 (66)	NS	446 (65)
N2	66 (33)	96 (39)	82 (34)		244 (35)
**Adjuvant chemotherapy,*n*(%)**					
Yes	140 (69)	169 (68)	125 (52)	< 0.01	434 (63)
No	62 (31)	79 (32)	115 (48)		256 (37)
**Timing of adjuvant chemotherapy,*n*(%)**					
< 8 weeks	59 (42)	72 (43)	36 (29)	< 0.05	167 (38)
> 8 weeks	81 (58)	97 (57)	89 (71)		267 (62)
**ACCI,*n*(%)**					
0–2	89 (44)	104 (42)	69 (29)	< 0.01	262 (38)
3–5	107 (53)	127 (51)	142 (59)		376 (54)
6–9	6 (3)	17 (7)	29 (12)		52 (8)
**Clavien-Dindo,*n*(%)**					
0–3a	202 (100)	239 (96)	203 (85)	< 0.01	644 (93)
3b–4	–	9 (4)	37 (15)		46 (7)
**Readmission**^b^**,*n*(%)**					
No	189 (94)	229 (92)	225 (94)	NS	643 (93)
Yes	13 (6)	19 (8)	15 (6)		47 (7)

Percentages within the parentheses should be read vertically, representing the top subject

*Abbreviations*: *IQR* interquartile range, *ACCI* age-adjusted Charlson Comorbidity Index, *NS* not significant.

^a^Percentage does not add up due to rounding

^b^Within 30 days

### Clinical characteristics related to length of stay

As shown in Table 1, the tumor-specific factors did not significantly differ between the LOS groups. Also, there was no difference in readmission rates between the different LOS groups and neither did comorbidity or age impact the risk of readmission. Though, patients with major complications (Clavien-Dindo 3b–4) had a higher rate of readmissions, 15% compared to 6% (*p* < 0.05).

Comorbidity and major postoperative complications were more common in the longer LOS group. Patients in the high comorbidity group (ACCI 6–9) had a mean LOS of 11.8 days compared to 7.9 days in the group with no or little comorbidity (ACCI 0–2) (*p* < 0.01). As expected, the 46 cases with major postoperative complications (Clavien-Dindo 3b–4) had a significantly longer LOS compared to those with no or minor complications (Clavien-Dindo 0–3a) (*p* < 0.01).

The median LOS in the hospital was 7 days with no difference over the years studied (*p* > 0.05) despite increased utilization of laparoscopic surgery during the years. Only 2 out of 42 patients (5%) had laparoscopic surgery in the year 2000 compared to 26 out of 56 (46%) in 2015 (*p* < 0.01). However, the frequency of T4 tumors increased, involving only 5 out of 42 (12%) operated tumors in 2000 to 14 out of 56 in 2015 (25%) (*p* < 0.01). There was no statistically significant difference in age or comorbidity (ACCI) over the studied period (*p*> 0.05).

### Factors influencing adjuvant chemotherapy

Fewer patients in the group with prolonged LOS received adjuvant chemotherapy (< 0.01, Table 1). Conversely, patients who received adjuvant chemotherapy had a shorter LOS (*p* < 0.01, Table 2). In total, 434 patients (63%) received adjuvant chemotherapy, where 69% of these received a single treatment with 5-fluorouracil (5-FU) together with leucovorin (FLV) and 31% were treated with a combination chemotherapy. The time from surgery to the initiation of adjuvant chemotherapy varied between the different LOS groups (*p*<0.01). The proportion of patients in the long LOS group with a late start (> 8 weeks) of adjuvant chemotherapy was higher (*p* < 0.05), and patients with a late start also had a longer mean LOS (*p* < 0.01, Additional file 1: Table S1).

**Table 2:** Clinical characteristics of stage III colon cancer patients by adjuvant chemotherapy

Variables	Adjuvant chemotherapy		***p***-value	Total (***n*** = 690)
	Yes (***n*** = 434)	No (***n*** = 256)		
**Hospital length of stay, days**				
Median (IQR)	7 (5–9)	8 (6–13)	< 0.01	7 (5–10)
Mean (SD)	8.0 (5.1)	10.2 (6.8)	< 0.01	9 (6)
**Age, median (min-max)**	65 (19–83)	81 (41–93)	< 0.01	71 (19–93)
**Gender,*n*(%)**				
Female	221 (51)	154 (60)	< 0.05	375 (54)
Male	213 (49)	102 (40)		315 (46)
**Surgery,*n*(%)**				
Open	368 (85)	217 (85)	NS	585 (85)
Laparoscopic	66 (15)	39 (15)		105 (15)
**Tumor location,*n*(%)**^a^				
Right sided	216 (50)	152 (59)	NS	368 (53)
Left sided	212 (49)	101 (39)		313 (45)
Multiple locations	6 (1)	3 (1)		9 (1)
**Differentiation grade,*n*(%)**				
Low grade (G1–G2)	308 (71)	175 (68)	NS	483 (70)
High grade (G3–G4)	105 (24)	63 (25)		168 (24)
Mucinous	21 (5)	18 (7)		39 (6)
**Number of positive lymph nodes, median (IQR)**	3 (1–5)	2 (1–4)	< 0.01	2 (1–5)
**T-stage,*n*(%)**				
1	8 (2)	2 (1)	< 0.05	10 (1)
2	28 (6)	16 (6)		44 (6)
3	291 (67)	196 (77)		487 (71)
4	107 (25)	42 (16)		149 (22)
**N-stage,*n*(%)**				
N1	268 (62)	178 (70)	< 0.05	446 (65)
N2	166 (38)	78 (30)		244 (35)
**ACCI,*n*(%)**^a^				
0–2	250 (58)	12 (5)	< 0.01	262 (38)
3–5	176 (41)	200 (78)		376 (54)
6–9	8 (2)	44 (17)		52 (8)
**Clavien-Dindo,*n*(%)**				
0–3a	407 (94)	237 (93)	NS	644 (93)
3b–4	27 (6)	19 (7)		46 (7)
**Readmission,*n*(%)**				
No	403 (93)	240 (94)	NS	643 (93)
Yes	31 (7)	16 (6)		47 (7)

Percentages within the parentheses should be read vertically, representing the top subject

*Abbreviations*: *IQR* interquartile range, *ACCI* age-adjusted Charlson Comorbidity Index, *NS* not significant

^a^Percentage does not add up due to rounding

Patients who received adjuvant chemotherapy were younger (*p* < 0.01, Table 2). In the age group 60 years or younger, 94% received adjuvant treatment, 88% among patients aged 61–70, and 35% in patients 71 years or older (*p* < 0.01). Among patients with no or low comorbidity (ACCI 0–2), 95% received adjuvant chemotherapy, 47% among patients with intermediate comorbidity (ACCI 3–5), and 15% in the high comorbidity group (ACCI 6–9) (*p* < 0.01). Major postoperative complications did not affect whether the patients received adjuvant chemotherapy, neither was there a difference in single or combination treatment in relation to major postoperative complications (Clavien-Dindo 0–3a or 3b–4).

The frequency of combination chemotherapies carried out and the timing of the adjuvant treatment changed during the studied period (*p* < 0.01). Only 4 out of 26 (15%) received combination chemotherapy in 2000 and 12 (46%) within 8 weeks. In 2015, 15 out of 33 (45%) received combination chemotherapy and 32 (97%) within 8 weeks. In total, more patients received combination therapy starting within 8 weeks compared to those who started after 8 weeks (*p* < 0.01, Additional file 1: Table S1).

### Postoperative hospital length of stay and outcomes

Among the 690 patients who underwent radical surgery for stage III colon cancer between the years 2000–2015, 34% (*n* = 234) relapsed in their disease, and 50% (*n* = 342) had any of the defined events (local or distal recurrence, all-cause mortality, or new CRC) (Table 3). Among the patients who had any of the defined events, the mean LOS was 10.0 days compared to 7.7 days in the censored group (*p* < 0.01). Fewer patients in the short and intermediate LOS groups had a relapse in their disease (28% and 33%, respectively), compared to the patients with longer LOS (40%, *p* < 0.05). There was no statistically significant difference in terms of postoperative complications or comorbidity as to whether the patients had a relapse in their disease or not.

**Table 3:** Clinical characteristics of stage III colon cancer patients by disease recurrence

Variables	Disease recurrence		***p***-value	Total (***n*** = 690)
	Yes (***n*** = 234)	No (***n*** = 456)		
**Hospital length of stay, days**				
Median (IQR)	7.5 (6–11)	7 (5–10)	< 0.05	7 (5–10)
Mean (SD)	9.5 (6.6)	8.5 (5.5)	< 0.05	
**Age, median (min-max)**	70 (25–93)	71 (19–92)	NS	71 (19–93)
**Gender,*n*(%)**				
Female	121 (52)	254 (56)	NS	375 (54)
Male	113 (48)	202 (44)		315 (46)
**Surgery,*n*(%)**				
Open	205 (88)	380 (83)	NS	585 (85)
Laparoscopic	29 (12)	76 (17)		105 (15)
**Tumor location,*n*(%)**				
Right sided	121 (52)	247 (54)	NS	368 (53)
Left sided	110 (47)	203 (45)		313 (45)
Multiple locations	3 (1)	6 (1)		9 (1)
**Differentiation grade,*n*(%)**				
Low grade (G1–G2)	161 (69)	322 (71)	NS	483 (70)
High grade (G3–G4)	59 (25)	109 (24)		168 (24)
Mucinous	14 (6)	25 (5)		39 (6)
**Number of positive lymph nodes, median (IQR)**	3.5 (2–6)	2 (1–4)	< 0.01	2 (1–5)
**T-stage,*n*(%)**				
1	0 (0)	10 (2)	< 0.01	10 (1)
2	8 (3)	36 (8)		44 (6)
3	164 (70)	323 (71)		487 (71)
4	62 (27)	87 (19)		149 (22)
**N-stage,*n*(%)**				
N1	117 (50)	329 (72)	< 0.01	446 (65)
N2	117 (50)	127 (28)		244 (35)
**ACCI,*n*(%)**^a^				
0–2	84 (36)	178 (39)	NS	262 (38)
3–5	137 (59)	239 (52)		376 (54)
6–9	13 (6)	39 (9)		52 (8)
**Clavien-Dindo,*n*(%)**				
0–3a	216 (92)	428 (94)	NS	644 (93)
3b–4	18 (8)	28 (6)		46 (7)
**Readmission,*n*(%)**				
No	219 (94)	424 (93)	NS	643 (93)
Yes	15 (6)	32 (7)		47 (7)

Percentages within parentheses should be read vertically, representing the top subject

*Abbreviation*: *IQR* interquartile range, *ACCI* Age–Adjusted Charlson comorbidity index, *NS* not significant

^a^Percent do not add up due to rounding

Furthermore, patients in the short and intermediate LOS groups were less likely to have any of the defined events (38% and 46%, respectively), compared to the patients with longer hospital LOS (63%, *p* < 0.01). Patients who had a relapse in their disease also had a longer mean LOS than patients without relapse, 9.5 days compared to 8.5 days (*p* < 0.05). Out of the whole cohort, 11% (*n* = 76) relapsed within 1 year from surgery. These patients had a mean LOS of 10.4 days, compared to 9.1 days among patients relapsing after 1 year and 8.5 days in patients who remained relapse-free (*p* < 0.05).

**Fig. 1:**
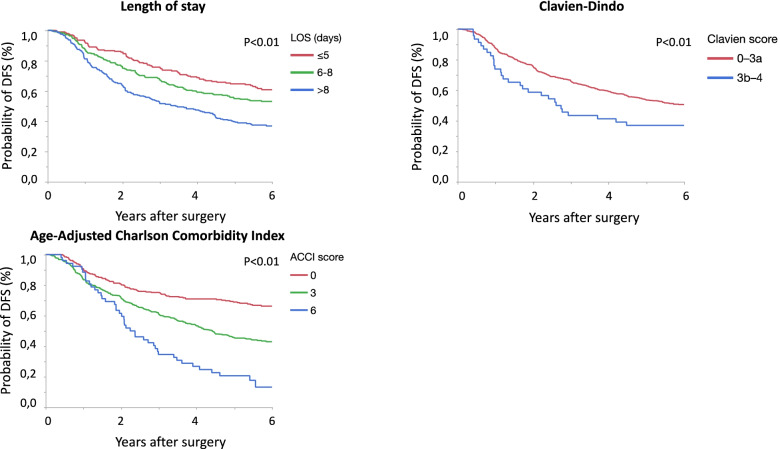
Disease-free survival among patients operated electively for stage III colon cancer between the years 2000–2015, in relation to length of stay, Clavien-Dindo, and age-adjusted Charlson Comorbidity Index

Patients with a short postoperative hospital LOS, low comorbidity, and few complications had a longer unadjusted DFS (*p* < 0.01, Fig. 1). The cohort with prolonged LOS had significantly shorter DFS compared to the cohorts with short and intermediate LOS (unadjusted HR 2.10 and 1.58, respectively, *p* < 0.01). Furthermore, without categorizing, postoperative hospital LOS was a significant prognostic factor for DFS when considered as a continuous variable in the unadjusted univariate model (HR 1.04 per unit change, Table 4). In the adjusted multivariate analysis, LOS remained statistically significant, with a HR of 1.03 per hospital day (*p* < 0.01). Comorbidities were also associated with a higher risk of the events (HR 1.59 in ACCI 3–5 and 2.40 in ACCI 6–9). Although significant in the univariate analysis, major postoperative complications (Clavien-Dindo grade) did not reach statistical significance in the multivariate model.

**Table 4:** Complete case multivariate Cox proportional hazards model for disease-free survival

Variables	Univariate			Multivariate		
	HR	95% CI	*p*	HR	95% CI	*p*
**Gender**						
Female	1					
Male	1.13	0.92–1.40	NS			
**Surgery**						
Open	1			1		
Laparoscopic	0.68	0.49–0.94	< 0.05	0.77	0.55–1.06	NS
**T-stage**						
T1–T3	1			1		
T4	1.33	1.04–1.71	< 0.05	1.26	0.98–1.63	NS
**N-stage**						
N1	1			1		
N2	1.59	1.28–1.97	< 0.01	1.60	1.29–2.00	< 0.01
**Differentiation**						
High (G1–G2)	1					
Low (G3–G4)	1.16	0.91–1.49	NS			
Mucinous	1.25	0.80–1.95	NS			
**Tumor location**						
Right sided	1					
Left sided	0.88	0.71–1.09	NS			
Multiple locations	0.75	0.28–2.03	NS			
**Adjuvant chemotherapy**						
Yes	1			1		
No	2.02	1.63–2.49	< 0.01	1.50	1.16–1.95	< 0.01
**Number of days in hospital**^a^	1.04	1.03–1.06	< 0.01	1.03	1.01–1.05	< 0.01
**Age-adjusted Charlson Comorbidity Index**						
0–2	1			1		
3–5	1.98	1.55–2.55	< 0.01	1.59	1.20–2.11	< 0.01
6–9	3.68	2.55–5.32	< 0.01	2.40	1.57–3.67	< 0.01
**Clavien-Dindo**						
0–3a	1			1		
3b–4	1.61	1.10–2.35	< 0.05	1.07	0.70–1.63	NS

Patients with a stage III colon cancer. Complete case multivariate Cox proportional hazards model (n=690) for disease free survival

*Abbreviation*: *NS* not significant

^a^Per unit change

**Fig. 2:**
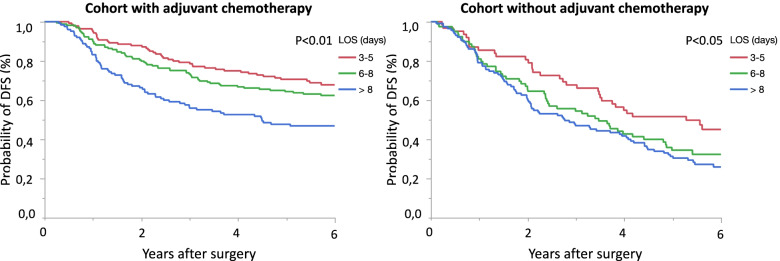
Disease-free survival among the three length of stay groups, presented with two survival plots: one cohort who received adjuvant chemotherapy and one who failed to receive adjuvant chemotherapy

A comparison of DFS among the LOS groups related to receiving adjuvant chemotherapy, in addition to no treatment at all, is shown in Fig. 2. As illustrated, DFS is shorter in the longer LOS groups, regardless of if adjuvant chemotherapy was given or not.

Receiving adjuvant chemotherapy after 8 weeks, compared to within 8 weeks, was associated with a shorter DFS (HR 1.55, *p* < 0.01). Patients who received adjuvant chemotherapy within 8 weeks after surgery had fewer recurrences compared to those who commenced later than 8 weeks, 28% and 37%, respectively (*p* < 0.05). However, no significant difference in DFS was shown once comparing adjuvant chemotherapy within or after 8 weeks, when distributed on the different LOS groups. Nevertheless, patients who did not receive adjuvant chemotherapy at all had a significantly shorter DFS in the longer LOS groups.

## Discussion

To our knowledge, this is the first study evaluating the association between prolonged postoperative hospital LOS and shorter DFS among patients undergoing radical resection of stage III colon cancer. In this detailed assessment, we have excluded confounding factors such as rectal tumors, other stages, emergency surgery, and postoperative mortality.

There are several studies conducted on what causes prolonged LOS in colorectal patients and its relations to other factors, such as comorbidity and complications. However, there are very few reports on how longer LOS affects the oncological outcome after surgery for colon cancer. Tartter concluded in 1996 that longer postoperative hospital LOS led to impaired survival for patients undergoing surgery for colorectal cancer [12]. Similarly, Shin et al. reported in 2017 that reduced postoperative hospital LOS led to improved survival among patients aged ≥ 70 years [13]. Within this study, we intentionally excluded rectal cancer patients whose LOS is known to be longer [7, 16]. Another unique characteristic of the present study is that we have put LOS in relation to adjuvant chemotherapy, only including stage III since the benefit of adjuvant chemotherapy is greater among this group in contrast to CRC at large [3, 4].

Many reports have concluded the association between delayed initiation of adjuvant treatment and a decreased OS and DFS [9, 10, 17], supporting prolonged LOS as an independent risk factor, since longer LOS after surgery causes delayed commencement of adjuvant chemotherapy [11]. Our study confirmed that an extended period from surgery to adjuvant treatment increased the risk of a shorter DFS, as well as the association between longer LOS and delayed initiation. Furthermore, patients with a prolonged LOS were less likely to receive adjuvant treatment compared to those with a shorter stay. As expected, adjuvant chemotherapy increased DFS in our study—however, prolonged LOS was still associated with a shorter DFS among patients who did not receive adjuvant treatment at all. Hence, it can be assumed that the shorter DFS among patients with longer LOS is not solely explained by delay or failure to receive adjuvant chemotherapy.

Previous studies have reported postoperative complications to be associated with a higher risk of recurrences and a worse overall survival, among patients undergoing surgery for colorectal cancer [18–20], and an especially strong relationship with anastomotic leakage [21–23]. Similar associations have been made for comorbidity, where cancer-specific survival has shown to be shorter [24, 25]. In the present study, both major postoperative complications and comorbidity were associated with a shorter DFS in the univariate analysis, but the results were not maintained for postoperative complications in the multivariate analysis. Neither did we find any correlation between postoperative complications and an increased risk for recurrence of the disease. This could perhaps partly be explained by the fact that the patients with postoperative complications received adjuvant chemotherapy to the same extent as those with a complication-free hospital stay. However, it may also be due to the relatively small sample size of major complications. In any case, comorbidity and postoperative complications cannot help to explain the elevated risk of recurrence we found among patients with a prolonged LOS, even though these variables were associated with a longer LOS. From the results, it is conceivable that a prolonged LOS not only leads to a higher risk but also to early recurrence.

There are some limitations to this study. Despite being conducted in an era where enhanced recovery protocols were internationally recognized, ERAS was not implemented at the studied hospital unit until 2016, perhaps explaining why there was no significant variation in LOS over the period of this study. Furthermore, laparoscopy was rarely carried out in the early years of this material, while almost half of the patients underwent laparoscopic resection at the end of the studied period. The laparoscopic group also had a better outcome in the unadjusted analyses, which might partly be attributed to the fact that they also had a significantly shorter LOS. Nevertheless, other confounding factors involved could simultaneously explain the generally improved oncological outcome in the latter years of the material. For example, during the studied period between 2000 and 2015, the adjuvant treatment regimens have been updated with the addition of oxaliplatin—combined with 5-FU—resulting in fewer combination therapies earlier on in the present study. Likewise, the time from surgery to the initiation of adjuvant chemotherapy has shortened, where more patients received adjuvant chemotherapy within 8 weeks later in the study. Faster turnaround time for the pathology reports and new recommendations of earlier commencement could be two possible explanations for this.

How is the reduced survival rate among patients with a longer postoperative stay explained, if not entirely by increased complications, age, comorbidity, or exclusion from adjuvant treatment? Rather than seeking for an explanation to the provided results, our objective was to evaluate if LOS had prognostic value in itself. One possible mechanism could be related to unidentified tumor immunological factors, where patients with a prolonged LOS have a reduced physical defense against cancer recurrence, perhaps due to inactivity in the postoperative phase which affects the rehabilitation negatively. Patients with prolonged LOS could also be more susceptible to nosocomial infections, which in turn might affect the immunological response. Both experimental and clinical studies on immunosuppression and progression of minimal residual disease in the postoperative phase after cancer surgery supports this theory [26, 27]. However, further studies are warranted to establish a direct causality.

## Conclusions

In conclusion, this study provides the first comprehensive assessment of postoperative hospital LOS as an independent prognostic factor for DFS among stage III colon cancer patients. In addition, we have demonstrated that patients with a recurrence in their disease had a longer postoperative stay. We believe that LOS represents a patient factor which is associated with DFS regardless of age, comorbidity, postoperative complications, and the initial tumor load. The results from this study could stress the importance of not excluding patients from adjuvant treatment because of prolonged LOS followed by a postponed follow-up. Without providing an explanation for these new discoveries, we recommend that prolonged LOS should be taken into consideration in the risk assessment of stage III colon cancer patients.

## Supplementary Information

**Additional file 1: Table S1**. Clinical characteristics of stage III colon cancer patients by timing of adjuvant chemotherapy (< 8 weeks or > 8 weeks).

## Data Availability

The datasets used and/or analyzed during the current study are available from the corresponding author upon reasonable request.

## Abbreviations

5-FU: 5-Fluorouracile
CCI: Charlson Comorbidity Index
CRC: Colorectal cancer
DFS: Disease-free survival
ERAS: Enhanced recovery after surgery
FLV: 5-FU and leucovorine
IQR: Interquartile range
LOS: Length of stay
NS: Not significant
OS: Overall survival
