# Supplementary material for: Prolonged postoperative length of stay may be a valuable marker for susceptibility to relapse beyond established risk factors in patients with stage III colon cancer
Source: World J Surg Oncol. 2022 Sep 2;20:277. doi: 10.1186/s12957-022-02742-8 (PMC9438186; doi:10.1186/s12957-022-02742-8)
Supplement: Supplementary file 1 — Additional file 1: Table S1. Clinical characteristics of stage III colon cancer patients by timing of adjuvant chemotherapy (< 8 weeks or > 8 weeks). [file 12957_2022_2742_MOESM1_ESM.docx]

| **Supplementary table 1**  Clinical characteristics of stage III colon cancer patients by timing of adjuvant chemotherapy (<8 weeks or >8 weeks) | | | | | | | | | | |
| --- | --- | --- | --- | --- | --- | --- | --- | --- | --- | --- |
|  | **Variables** |  | **Timing of adjuvant chemotherapy:** | | |  |  |  |  | |
|  |  |  | **≤8 weeks (*n=*167*)*** |  | **>8 weeks (*n=*267)** |  | ***p–value*** |  | **Total (*n*= 434)** |  |
|  |  |  |  |  |  |  |  |  |  |  |
|  | **Hospital length of stay** |  |  |  |  |  |  |  |  |  |
|  | Median (IQR) |  | 6 (5–8) |  | 7 (5–10) |  | <0.05 |  | 7 (5–9) |  |
|  | Mean (SD) |  | 7.2 (3.6) |  | 8.6 (5.8) |  | <0.05 |  | 8 (5) |  |
|  | **LOS-cohorts**, n (%)* |  |  |  |  |  |  |  |  |  |
|  | ≤5 days |  | 59 (35) |  | 81 (30) |  | <0.05 |  | 140 (32) |  |
|  | 6-8 days |  | 72 (43) |  | 97 (36) |  |  |  | 169 (39) |  |
|  | >8 days |  | 36 (22) |  | 89 (33) |  |  |  | 125 (29) |  |
|  | **Age,** median (Min–max) |  | 61 (19–80) |  | 68 (19–83) |  | <0.01 |  | 65 (19–83) |  |
|  | **Gender**, n (%) |  |  |  |  |  |  |  |  |  |
|  | Female |  | 91 (54) |  | 130 (49) |  | NS |  | 221 (51) |  |
|  | Male |  | 76 (46) |  | 137 (51) |  |  |  | 213 (49) |  |
|  | **Surgery**, n (%) |  |  |  |  |  |  |  |  |  |
|  | Open |  | 128 (77) |  | 240 (90) |  | <0.01 |  | 368 (85) |  |
|  | Laparoscopic |  | 39 (23) |  | 27 (10) |  |  |  | 66 (15) |  |
|  | **Tumor location**, n (%) |  |  |  |  |  |  |  |  |  |
|  | Right sided |  | 77 (46) |  | 139 (52) |  | NS |  | 216 (50) |  |
|  | Left sided |  | 86 (52) |  | 126 (47) |  |  |  | 212 (49) |  |
|  | Multiple locations |  | 4 (2) |  | 2 (1) |  |  |  | 6 (1) |  |
|  | **Differentiation grade,** n (%)* |  |  |  |  |  |  |  |  |  |
|  | Low grade (G1–G2) |  | 120 (72) |  | 188 (70) |  | NS |  | 308 (71) |  |
|  | High grade (G3–G4) |  | 36 (22) |  | 69 (26) |  |  |  | 105 (24) |  |
|  | Mucinous |  | 11 (7) |  | 10 (4) |  |  |  | 21 (5) |  |
|  | **Number of positive lymph nodes,** median (IQR) |  | 3 (1–6) |  | 3 (1–5) |  | NS |  | 3 (1–5) |  |
|  | **T–stage**, n (%) |  |  |  |  |  |  |  |  |  |
|  | 1 |  | 3 (2) |  | 5 (2) |  | NS |  | 8 (2) |  |
|  | 2 |  | 12 (7) |  | 16 (6) |  |  |  | 28 (6) |  |
|  | 3 |  | 102 (61) |  | 189 (71) |  |  |  | 291 (67) |  |
|  | 4 |  | 50 (30) |  | 57 (21) |  |  |  | 107 (25) |  |
|  | **N–stage**, n (%) |  |  |  |  |  |  |  |  |  |
|  | N1 |  | 103 (62) |  | 165 (62) |  | NS |  | 268 (62) |  |
|  | N2 |  | 64 (38) |  | 102 (38) |  |  |  | 166 (38) |  |
|  | **ACCI**, n (%) * |  |  |  |  |  |  |  |  |  |
|  | 0–2 |  | 118 (71) |  | 132 (49) |  | <0.01 |  | 250 (58) |  |
|  | 3–5 |  | 48 (29) |  | 128 (48) |  |  |  | 176 (41) |  |
|  | 6–9 |  | 1 (1) |  | 7 (3) |  |  |  | 8 (2) |  |
|  | **Clavien–Dindo**, n (%) |  |  |  |  |  |  |  |  |  |
|  | 0–3a |  | 160 (96) |  | 247 (93) |  | NS |  | 407 (94) |  |
|  | 3b–4 |  | 7 (4) |  | 20 (7) |  |  |  | 27 (6) |  |
|  | **Readmission**, n (%)***** |  |  |  |  |  |  |  |  |  |
|  | No |  | 158 (95) |  | 245 (92) |  | NS |  | 403 (93) |  |
|  | Yes |  | 9 (5) |  | 22 (8) |  |  |  | 27 (6) |  |
|  | **Type of chemotherapy,** n (%) |  |  |  |  |  |  |  |  |  |
|  | Single |  | 96 (57) |  | 201 (75) |  | <0.01 |  | 297 (68) |  |
|  | Combination |  | 71 (43) |  | 66 (25) |  |  |  | 137 (32) |  |
|  | | | | | | | | | | |
| Percentages within parentheses should be read vertically, representing the top subject. | | | | | | | | | | |
| Abbreviation: *LOS,* length of stay; *IQR,* interquartile range; *ACCI*, age-adjusted charlson comorbidity index; *NS*, not significant. *Percent do not add up due to rounding. | | | | | | | | | | |
